# A review of the spider genus *Sinanapis*, with the description of a new species from Tibet (Araneae, Anapidae)

**DOI:** 10.3897/zookeys.790.25793

**Published:** 2018-10-15

**Authors:** Qiqi Zhang, Yucheng Lin

**Affiliations:** 1 Key Laboratory of Bio-resources and Eco-environment (Ministry of Education), College of Life Sciences, Sichuan University, Chengdu, Sichuan 610064, China Sichuan University Chengdu China

**Keywords:** Araneoidea, anapids, Asia, key, revision, Xizang

## Abstract

The genus *Sinanapis* Wunderlich & Song, 1995 is reviewed in this paper. The material of all three known species was reexamined and photographed resulting in a new species, *Sinanapismedogense***sp. n.** (♂, ♀) being described from Tibet, China. A key is provided for the genus, as well as species diagnoses, illustrations, and distribution maps for all four species of *Sinanapis*.

## Introduction

According to the World Spider Catalog (2018), 223 extant species in 58 genera are documented in the family Anapidae Simon, 1895, including eleven species in seven genera from China. This family is chiefly distributed in the tropical and southern temperate regions (Lin and Li 2012).

The *Sinanapis* was originally erected by Wunderlich and Song (1995) as a monotypic genus based on *S.crassitarsa* Wunderlich & Song, 1995 from Xishuangbanna in Yunnan of China. Currently *Sinanapis* comprises three valid species distributed in southern China, Vietnam and Laos: *S.crassitarsa* Wunderlich & Song, 1995, *S.longituba* Lin & Li, 2012, and *S.wuyi* Jin & Zhang, 2013, making *Sinanapis* the genus with the highest number of species within the family Anapidae in China. The genus was previously known in China from Yunnan to Fujian Provinces only.

While studying material from Tibet, we recognized several specimens belonging to Anapidae. Detailed study of these specimens reveals that they belong to an undescribed species of *Sinanapis*, a genus previously unknown in Tibet. The goal of this paper is to provide detailed description of the new species and to conduct a comprehensive review of the genus *Sinanapis*.

## Materials and methods

Specimens were examined and measured with a Leica M205 C stereomicroscope. Further details were studied with an Olympus BX43 compound microscope. Male and female copulatory organs were examined after they were dissected and detached from the bodies. Epigyne were removed and treated with lactic acid before photographed. All type specimens were preserved in 95% ethanol. Photos were taken with a Canon EOS 60D wide zoom digital camera (8.5 megapixels) mounted on an Olympus BX43 stereomicroscope. The images were montaged using Helicon Focus 3.10 (Khmelik et al. 2006) image stacking software.

All measurements are in millimeters. Leg measurements are given in the following sequence: total length (femur, patella, tibia, metatarsus, and tarsus). Abbreviations in figures or text are as follows:

**ALE** anterior lateral eyes;

**AME** anterior median eyes;

**BA** basal patellar apophysis on palp;

**BC** book lung covers;

**CD** copulatory ducts;

**Cu** cusps on leg I;

**Cy** cymbium;

**CO** copulatory opening;

**DA** dorsal patellar apophysis on palp;

**DP** dentigerous patellar process on palp;

**Em** embolus;

**FD** fertilization ducts;

**Fe** femur;

**LA** lateral patellar apophysis on palp;

**LS** labral spur;

**Pa** patella;

**PLE** posterior lateral eyes;

**PME** posterior median eyes;

**S** spermathecae;

**TA** tibial apophysis on palp;

**Ti** tibia;

**Te** tegulum.

All examined materials are deposited in the following institutions:

**SMF** Senchenberg Research Institete, Frankfurt, Gremany

**IZCAS** Institute of Zoology, Chinese Academy of Sciences in Beijing, China

**NHMSU** Natural History Museum of Sichuan University in Chengdu, China

**HNU** School of Life Sciences, Hunan Normal University in Changsha, China

**MHBU** Museum of Hebei University in Baoding, China

## Taxonomy

### Family Anapidae Simon, 1895

#### Genus Sinanapis

Taxon classificationAnimaliaAraneaeAnapidae

Wunderlich & Song, 1995

##### Type species.

*Sinanapiscrassitarsa* Wunderlich & Song, 1995 from Xishuangbanna, Yunnan.

##### Diagnosis.

**Figure 1.**
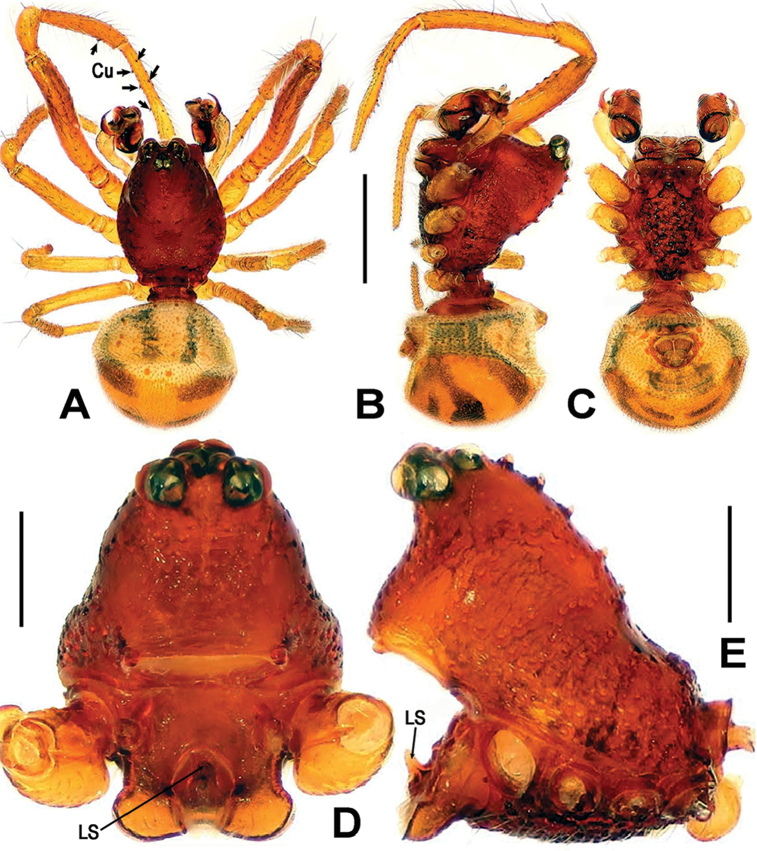
*Sinanapiscrassitarsa* Wunderlich & Song, 1995, **A–C** Male habitus **D, E** Prosoma (chelicerae and appendages omitted) **A** dorsal **B, E** lateral **C** ventral **D** anterior. Abbreviations: **Cu** cusps on leg I; **LS** labial spur. Scale bars: 0.50 (**A–C**); 0.20 (**D, E**).

**Figure 2.**
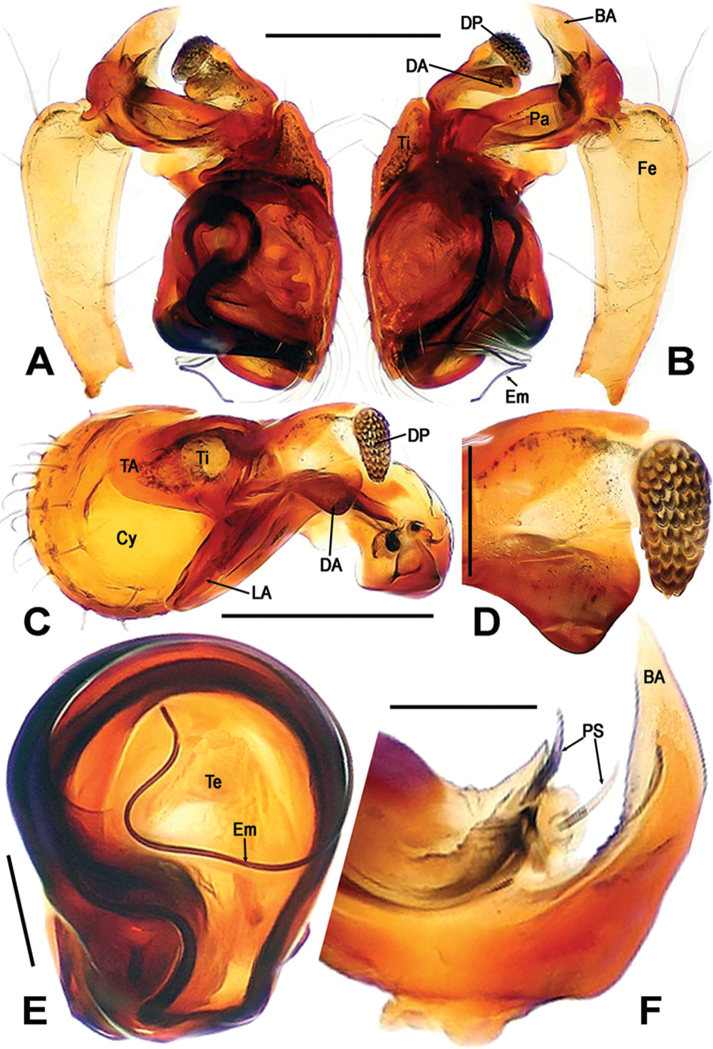
*Sinanapiscrassitarsa* Wunderlich & Song, 1995, **A–C** Male left palp **D** Dorsal patellar apophyses on palp **E** Bulb **F** Basal patellar apophysis **A** prolateral **B, F** reterolateral **C, D** dorsal **E** ventral. Abbreviations: **BA** basal patellar apophysis; **Cy** cymbium; **DA** dorsal patellar apophysis; **DP** dentigerous patellar process; **Em** embolus; **Fe** femur; **LA** lateral patellar apophysis; **Pa** patella; **PS** patellar spine; **TA** tibial apophysis; **Te** tegulum; **Ti** tibia. Scale bars: 0.20 (**A–C**); 0.10 (**D, E**); 0.05 (**F**).

The males of *Sinanapis* can be distinguished from other male anapids by the palp with at least 3 patellar apophyses (Figs 2C, 4A, 6H, 9D), the ventrally flat bulb lacking conductor (Figs 2E, 4B, 6E, 9C), the embolus coiling around the bulb margin in at least one loop (Figs 2E, 4B, 6E, 9C), and having ventral cusps on metatarsus and tarsus I (Figs 1A, 4D, 6D). Females of *Sinanapis* can be distinguished from other Chinese anapids by the globular spermathecae spaced by less than 1.5 diameters (Figs 4H, 7I, 9F), and the copulatory ducts with at least one loop (Figs 4H, 7I, 9F).

##### Composition.

*Sinanapiscrassitarsa* Wunderlich & Song, 1995 (♂), *S.longituba* Lin & Li, 2012 (♂, ♀), *S.medogense* sp. n. (♂, ♀), and *S.wuyi* Jin & Zhang, 2013 (♂, ♀).

##### Distribution.

China (Tibet, Yunnan, Hunan, Jiangxi, Fujian, Hainan), Laos, Vietnam.

##### Remarks.

This genus gender is considered as masculine at its establishment by Wunderlich and Song (1995). But it was later corrected to feminine by World Spider Catalog (2018).

### Key to species of *Sinanapis* Wunderlich & Song, 1995

**Table:** 

1	Males	**2**
–	Females	**5**
2	Anterior median eyes present (Fig. 3G); palp without a rasper-like dentigerous patellar process (Figs 4A, 9D)	**3**
–	Anterior median eyes absent (Figs 1D, 5G); palp with a rasper-like dentigerous patellar process (Figs 1C, 6H–I)	**4**
3	Leg I robust (Fig. 4C–D); basal patellar apophysis of palp very long, more than 3 times longer than patella; dorsal patellar apophysis long, narrow (Fig. 4A–B)	*** S. longituba ***
–	Leg I normal (Fig. 8A, D); basal patellar apophysis of palp not longer than patella; dorsal patellar apophysis short, wide (Fig. 9B, D)	*** S. wuyi ***
4	A rasper-like dentigerous patellar process as large as dorsal patellar apophysis (Fig. 2B–D); basal patellar apophysis short, laminar (Fig. 2F)	*** S. crassitarsa ***
–	A rasper-like dentigerous patellar process shorter than dorsal patellar apophysis (Fig. 6H–I); basal patellar apophysis long, tubular (Fig. 6B, G)	***S.medogense* sp. n.**
5	Anterior median eyes present (Fig. 3G)	**6**
–	Anterior median eyes absent (Figs 1D, 5G)	***S.medogense* sp. n.**
6	Abdomen with white pattern dorsally and laterally (Fig. 3C, F); copulatory duct wide, with 2 loops, coiled around the entire spermatheca (Fig. 4H)	*** S. longituba ***
–	Abdomen without white pattern dorsally and laterally (Fig. 8D, F); copulatory duct narrow, with one loop, coiled around the base of spermatheca (Fig. 9F)	*** S. wuyi ***

#### Sinanapis crassitarsa

Taxon classificationAnimaliaAraneaeAnapidae

Wunderlich & Song, 1995

Figs 1 , 2 Ono, 2009 (see Lin et al. 2013).

##### Type material.

Holotype ♂ (IZCAS), CHINA: Yunnan Province, Xishuangbanna Dai Autonomous Prefecture, Mengla County, Menglun Town, tropical botanical garden near rainforest, in leaf litter, 2.X.1987, L.M. Yu leg. (not examined).

##### Other material examined.

1♂ (SMF), LAOS: Champasak Province, Muang Bachieng, Ban Lak 35, That Etu, secondary forest, sieved leaf litter near waterfall, 15°11.628'N, 106°06.105'E; 595 m, 26-XI-2009, P. Jäger leg.

##### Diagnosis.

**Figure 3.**
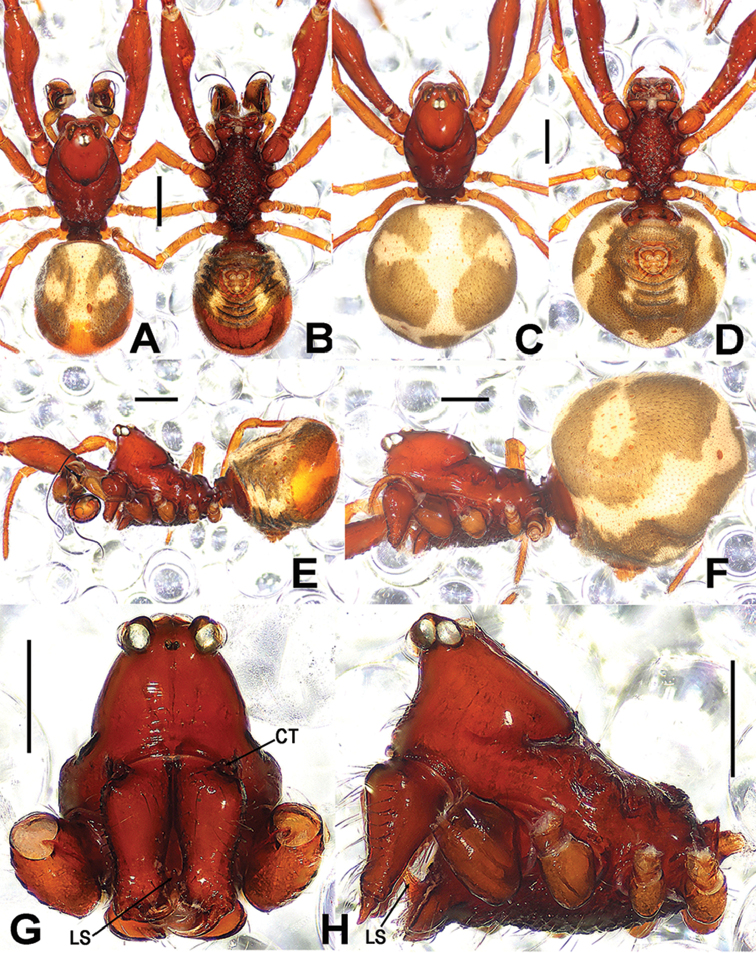
*Sinanapislongituba* Lin & Li, 2012, **A, B, E** Male habitus **C, D, F** Female habitus **G, H** Prosoma (appendages omitted) **A, C** dorsal **B, D** ventral **E, F, H** lateral **G** anterior. Abbreviations: **CT** cheliceral tubercle; **LS** labial spur. Scale bars: 0.50 (**A–F**); 0.20 (**G, H**).

**Figure 4.**
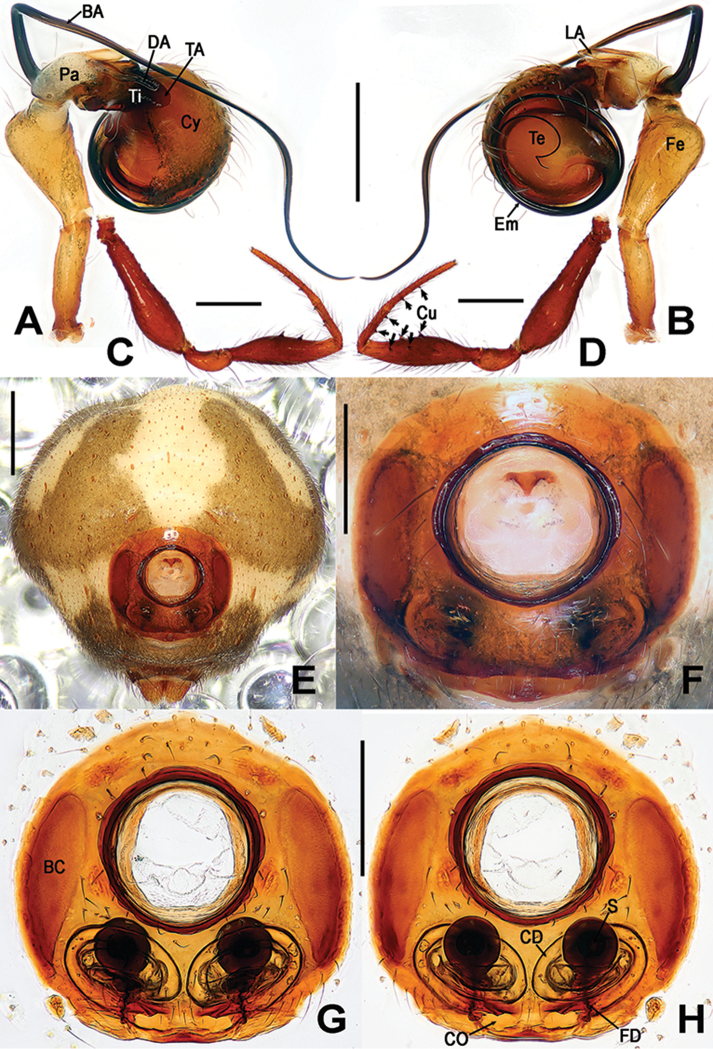
*Sinanapislongituba* Lin & Li, 2012, **A, B** Male left palp **C, D** Male leg I **E** Female abdomen **F**Epigyne**G, H** Vulva **A, D** prolateral **B, C** reterolateral **E–G** ventral **H** doral. Abbreviations: **BA** basal patellar apophysis; **BC** booklung cover; **CD** copulatory ducts; **CO** copulatory opening; **Cy** cymbium; **Cu** cusps on leg I; **DA** dorsal patellar apophysis; **Em** embolus; **FD** fertilization ducts; **Fe** femur; **LA** lateral patellar apophysis; **Pa** patella; **S** spermathecae; **TA** tibial apophysis; **Te** tegulum; **Ti** tibia. Scale bars: 0.20 (**A, B, E–H**); 0.50 (**C, D**).

*Sinanapiscrassitarsa* may be distinguished from the other two species except *S.medogense* sp. n. by having a rasper-like dentigerous patellar process and the absence of anterior median eyes (Figs 1D, 2C, D). In contrast, the other two species lack the dentigerous patellar process, and the anterior median eyes are present (Figs 3G, 4A, 9D). It may be distinguished from *S.medogense* sp. n. by the wide, laminar basal patellar apophysis, and the dentigerous patellar process is as large as the dorsal patellar apophysis (Figure 2B–D, F). In contrast, *S.medogense* sp. n. has a narrow, tubular basal patellar apophysis, and its dentigerous patellar process is smaller than dorsal patellar apophysis (Figure 6A, B, F–I).

##### Description.

See Figs 1A–E, 2A–F and Wunderlich and Song (1995), see also Song et al. (1999) and Lin et al. (2013).

##### Distribution.

China (Yunnan), Laos, and Vietnam.

#### Sinanapis longituba

Taxon classificationAnimaliaAraneaeAnapidae

Lin & Li, 2012

Figs 3 , 4

##### Type material.

*Holotype*: ♂ (IZCAS), CHINA: Hainan Province, Qiongzhong City, Mt. Limushan Nature Reserve, in leaf litter, 19°11.000'N, 109°44.000'E; 655 m, 12.VIII.2007, S.Q. Li & C.X. Wang leg. *Paratypes*: 3♂, 11♀ (IZCAS), same data as holotype (examined).

##### Other material examined.

4♂, 2♀ (NHMSU), CHINA: Hainan Province, Qiongzhong City, Yinggeling National Natural Reserve, Yinggezui Management Station, 19°03.037'N, 109°44.899'E; 622 m, 8–9.V.2011, Y.Y. Zhou leg.; 1♀ (NHMSU), CHINA: Hainan Province, Baisha County, Yuanmen Town, Hongxin Village, Yinggeling, 19°03.643'N, 109°31.329'E; 598±11 m, 27.III.2013, Z.G. Chen leg.

##### Diagnosis.

**Figure 5.**
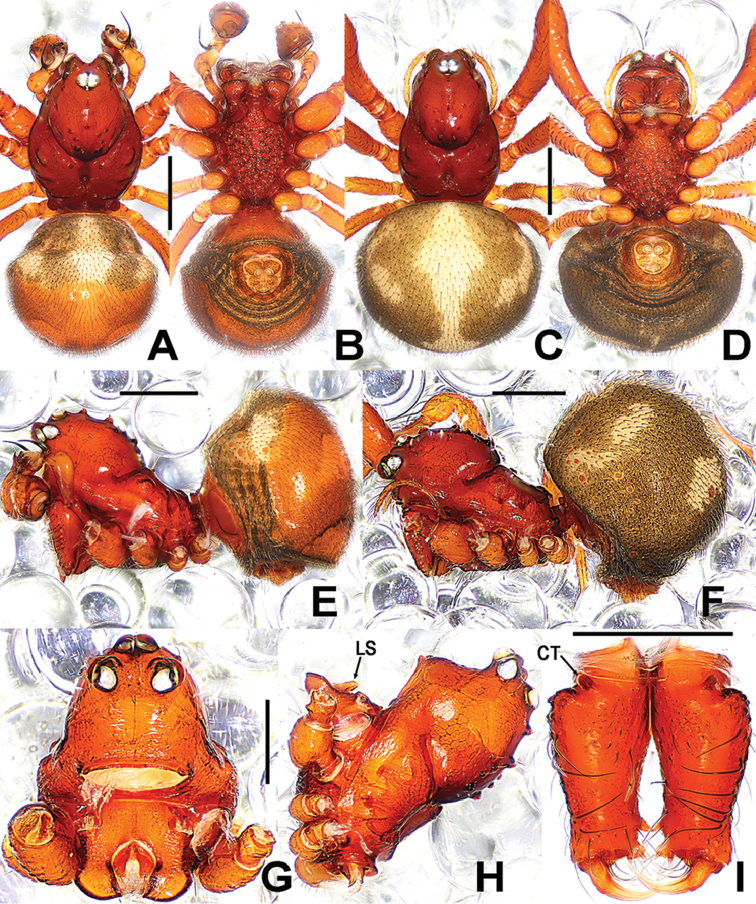
*Sinanapismedogense* sp. n., male holotype (**A–B, E, G–I**) and female paratype (**C–D, F**) from Xizang. **A–F** Habitus **G, H** Prosoma **I** Chelicerae **A, C** dorsal **B, D** ventral **E–F, H** lateral **G, I** frontal. Abbreviations: **CT** cheliceral tubercle; **LS** labial spur. Scale bars: 0.50 (**A–F**); 0.25 (**G–I**).

**Figure 6.**
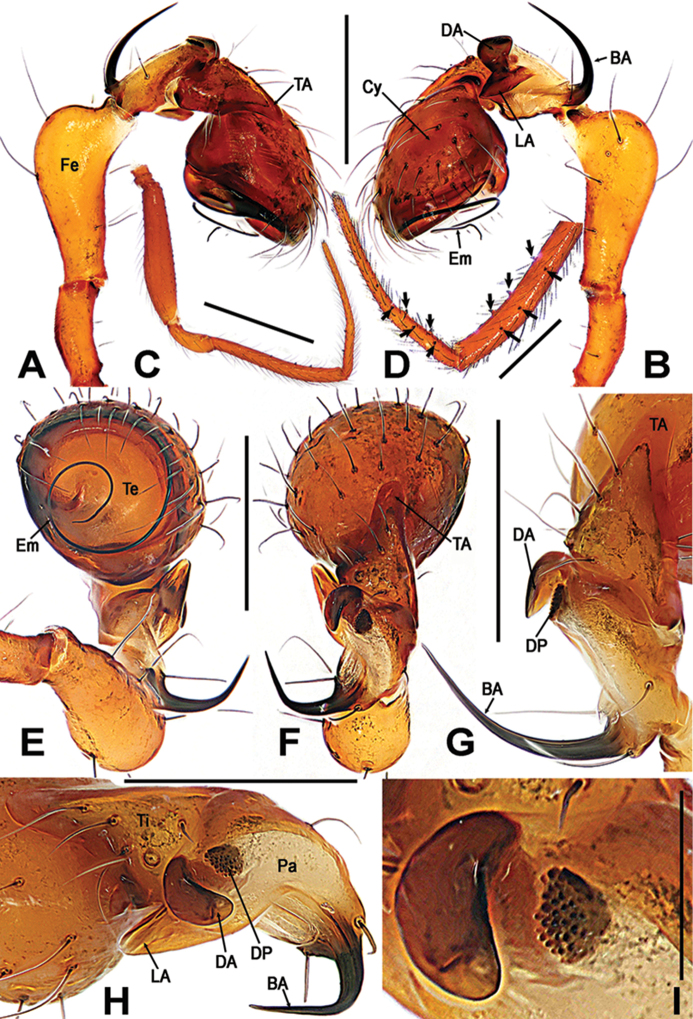
*Sinanapismedogense* sp. n., male holotype from Xizang. **A, B, E, F** Left palp **C, D** Left leg I **G, H** Palpal patella and tibia **I** Patellar apophysis **A, D, G** prolateral **B, C** retrolateral **E** ventral **F, H, I** dorsal. Abbreviations: **BA** basal patellar apophysis; **Cu** cusps on leg I; **Cy** cymbium; **DA** dorsal patellar apophysis; **DP** dentigerous patellar process; **Em** embolus; **Fe** femur; **LA** lateral patellar apophysis; **Pa** patella; **TA** tibial apophysis; **Ti** tibia; **Te** tegulum. Scale bars: 0.25 (**A, B, E–H**); 1.00 (**C**); 0.50 (**D**); 0.05 (**I**).

**Figure 7.**
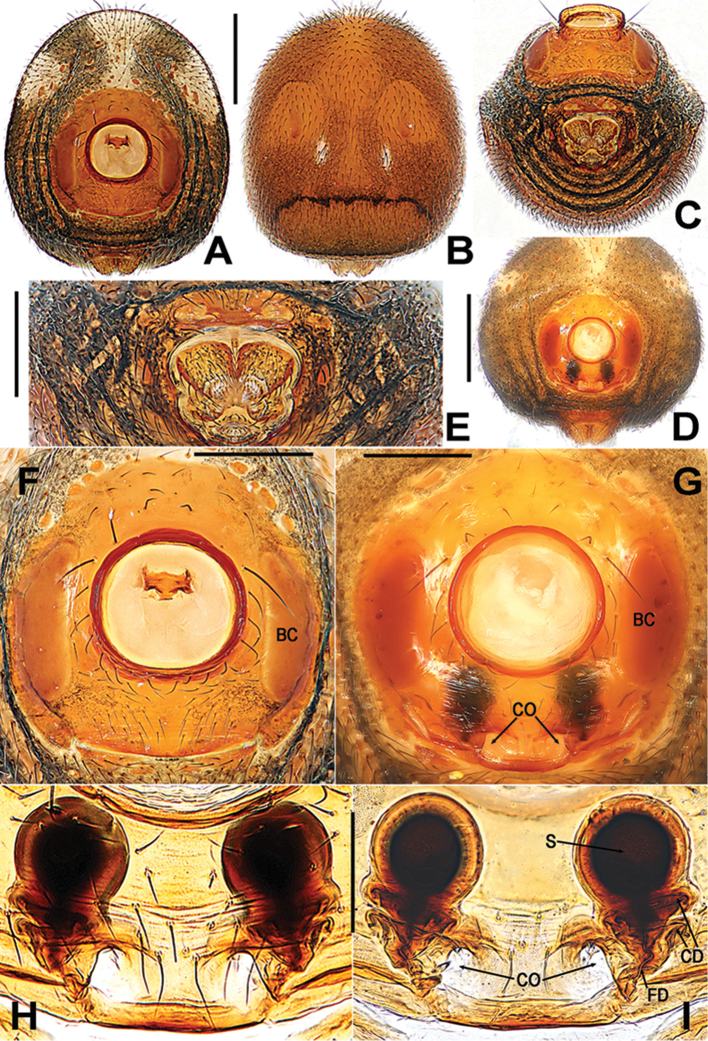
*Sinanapismedogense* sp. n., male holotype (**A–C, E, F**) and female paratype (**D, G–I**) from Xizang. **A–D** Abdomen **E** Spinnerets **F** Epigastric scutum **G**Epigyne**H, I** Vulva (lactic acid-treated) **A, D, F–H** ventral **B, I** dorsal **C, E** antapical. Abbreviations: **BC** booklung covers; **CD** copulatory duct; **CO** copulatory opening; **FD** fertilization duct; **S** spermatheca. Scale bars: 0.50 (**A–D**); 0.20 (**E–G**); 0.10 (**H, I**).

**Figure 8.**
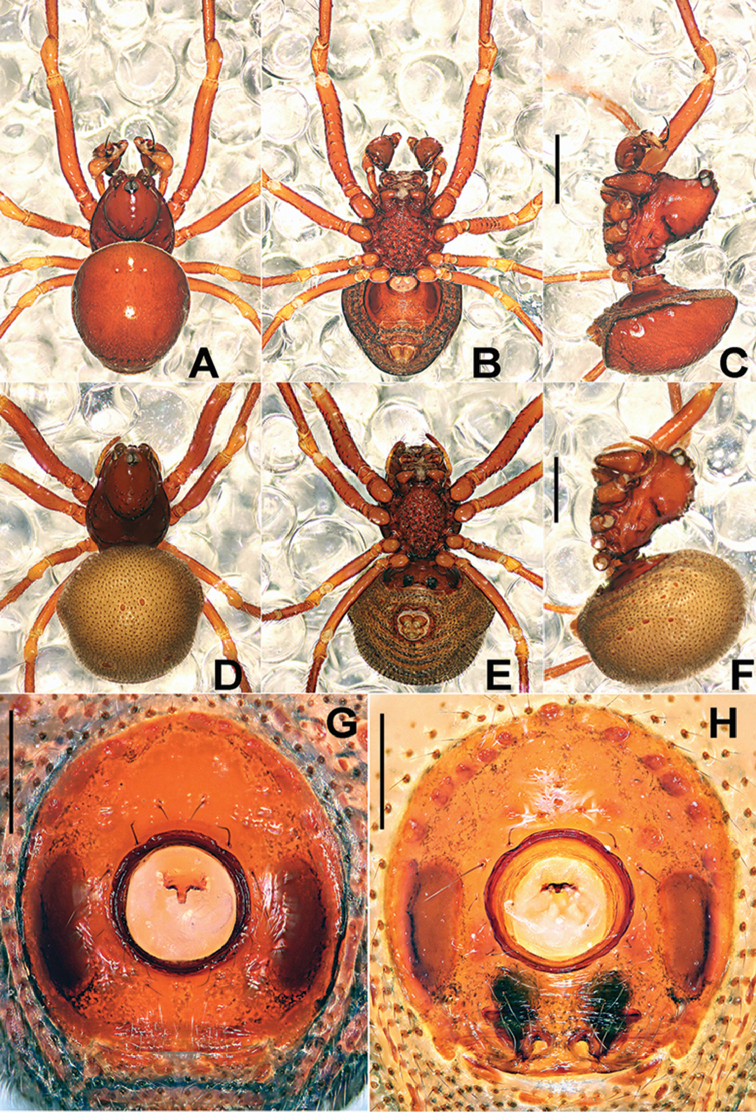
*Sinanapiswuyi* Jin & Zhang, 2013, male (**A–C, G**) and female (**D–F, H**) paratypes. **A–F** Habitus **G** Epigastric scutum **H**Epigyne**A, D** dorsal **B, E, G, H** ventral **C, F** lateral. Scale bars: 0.50 (**A–F**); 0.20 (**G, H**).

**Figure 9.**
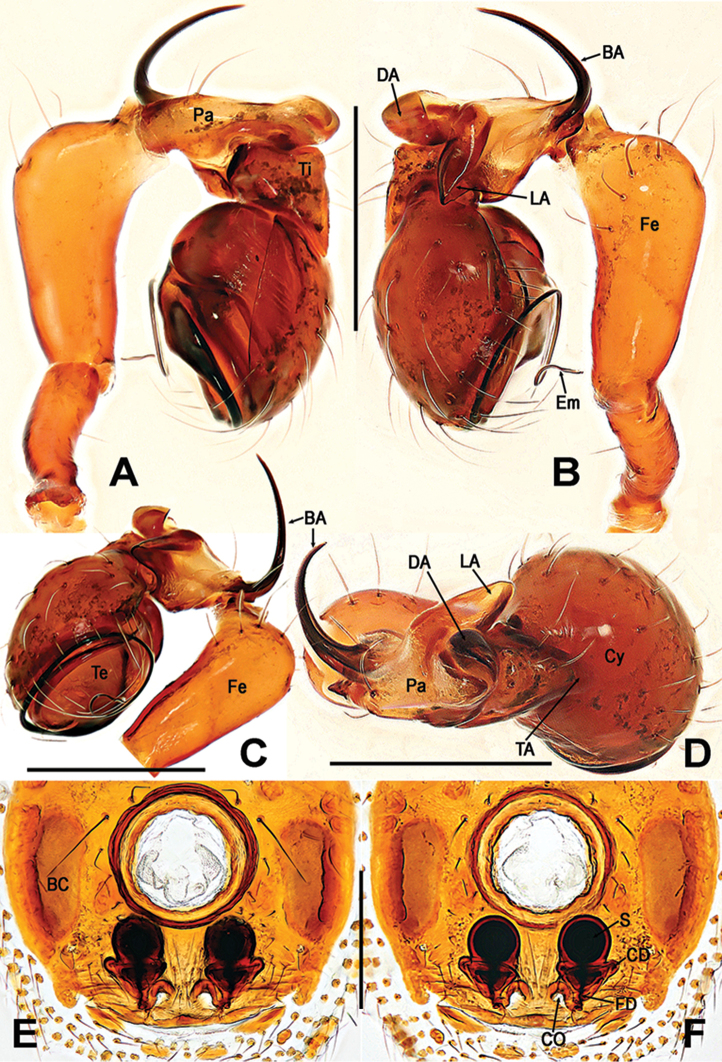
*Sinanapiswuyi* Jin & Zhang, 2013, male (**A–D**) and female (**E, F**) paratypes. **A–D** Left palp **E, F** Vulva **A** prolateral **B** retrolateral **C** retro-ventral **D, F** dorsal **E** ventral. Abbreviations: **BA** basal patellar apophysis; **BC** booklung cover; **CD** copulatory ducts; **CO** copulatory opening; **Cy** cymbium; **DA** dorsal patellar apophysis; **Em** embolus; **FD** fertilization ducts; **Fe** femur; **LA** lateral patellar apophysis; **Pa** patella; **S** spermathecae; **TA** tibial apophysis; **Te** tegulum; **Ti** tibia. Scale bars: 0.20 (**A–D**); 0.25 (**E, F**).

The male of *S.longituba* can be distinguished from *S.crassitarsa* and *S.medogense* sp. n. by the presence of anterior median eyes (Figure 3G), lacking in two latter species (Figs 1D, 5G), and by the absence of a rasper-like dentigerous process (Figure 3A, B), whereas the dentigerous process is present in the other two species (Figs 2C, 6H). It differs from *S.wuyi* by the robust leg I in both sexes (Figs 3A–D, 4C, D), as against the normal leg I seen in *S.wuyi* (Figure 8A, B, D, E). It further differs from *S.wuyi* by having a very long basal patellar apophysis, 3 times longer than palpal femur (Figure 4A, B), while the basal patellar apophysis is shorter than the palpal femur in *S.wuyi* (Figure 9A, B). The female of *S.longituba* can be distinguished from that of the congeners by the larger copulatory openings and the longer copulatory ducts around the spermathecae (Figure 4G, H). On the other hand, the copulatory openings are smaller in *S.medogense* sp. n. (Figure 7G, I) and *S.wuyi* (Figure 9E, F) and their shorter copulatory ducts do not around the spermathecae.

##### Description.

See Figs 3A–H, 4A–H and Lin and Li (2012).

##### Distribution.

China (Hainan).

#### Sinanapis medogense sp. n.

Taxon classificationAnimaliaAraneaeAnapidae

http://zoobank.org/DD8E8CB0-CB1B-4100-AD16-FAD82BCDCC83

Figs 5 , 6 , 7

##### Type material.

*Holotype*: ♂ (NHMSU), CHINA: Tibet Autonomous Region, Nyingchi Prefecture, Medog County, Renqinbeng Mountain, 29°19.050'N, 95°19.998'E; 1314 m, 26.VIII.2015, J.L. Wu leg. *Paratypes*: 1♂, 2♀ (NHMSU), same data as holotype.

##### Etymology.

The specific name derives from the type locality; adjective.

##### Diagnosis.

The male of this new species can be distinguished from that of *S.longituba* and *S.wuyi* by the lack of anterior median eyes and having a rasper-like dentigerous process (Figs 5G, 6G, H). In the case of the two latter species, the anterior median eyes are present, and the rasper-like dentigerous patellar process is absent (Figs 3G, 4A, 9D; Yuan and Peng, 2014: figs 7, 9). It also differs from *S.crassitarsa* by having a tubular basal apophysis, and a smaller dentigerous process (Figure 6A, B, G–I). In *S.crassitarsus*, the basal apophysis is laminar, and the dentigerous process is larger (Figure 2A–D, F). The female of the new species differs from *S.longituba* by having shorter copulatory ducts, each coiling with less than two loops next to the spermatheca (Figure 7I). In *S.longituba*, each copulatory coil around the spermatheca in more than two loops (Figure 4H). *S.medogense* further differs from *S.wuyi* by the absence of anterior median eyes, and by having a white pattern on the abdomen (Figure 5C, F), whereas the anterior median eyes are present and the abdominal white pattern is absent in *S.wuyi* (Figure 8D, F; Yuan and Peng, 2014: figs 7, 9).

##### Description.

**Male** (holotype): Somatic characters and coloration as in Figs 5A, B, E, G–I, 7A–C, E, F. *Measurements*: Total length 1.86. Carapace 0.96 long, 0.72 wide, 0.72 high. Clypeus 0.40 high. Sternum 0.52 long, 0.42 wide. Abdomen 0.90 long, 0.94 wide. Length of legs: I 3.76 (1.18, 0.40, 1.04, 0.42, 0.72); II 2.68 (0.82, 0.32, 0.62, 0.32, 0.60); III 2.02 (0.60, 0.22, 0.40, 0.28, 0.52); IV 2.52 (0.78, 0.24, 0.60, 0.34, 0.56).

*Palp* (Figure 6A–I): Trochanter very long, subequal to 2/3 of femur length. Femur distally swollen approx. 2 times wider than proximally. Patella, complex, each modified with four apophyses (Figure 6H): basal apophysis long horned, almost as long as patella; two dorsal apophyses, one crooked and fingerlike, and another rasper-like dentigerous process (Figure 6I); a lateral apophysis straight, finger-shaped, protruded. Tibia with a dorsal apophysis and a dorsal trichobothrium (Figure 6F–H). Cymbium bowl-shaped, as wide as long, covered with sparse long setae. Bulb simple, cone-shaped, tegulum smooth and flat, without any apophysis. Embolus long, strongly sclerotized, started at the middle margin of bulb, and ends in the above of subcentral bulb, coiled almost into two loops, distally tapering (Figure 6A, E).

**Female** (paratype). Somatic characters and coloration as in Figs 5C, D, F, 7D. *Measurements*: Total length 1.96. Carapace 1.02 long, 0.64 wide, 0.80 high. Abdomen 0.94 long, 0.43 wide. Clypeus 0.46 high. Sternum 0.61 long, 0.43 wide. Length of leg: I 3.42 (1.12, 0.36, 0.90, 0.40, 0.64); II 2.48 (0.76, 0.30, 0.58, 0.28, 0.56); III 1.8 (0.54, 0.20, 0.38, 0.22, 0.46); IV 2.3 (0.72, 0.24, 0.56, 0.28, 0.50).

*Epigyne* (Figure 7G–I): Epigyne sclerotized, almost rectangular, about 2 times wider than booklung cover, vulva visible through the translucent integument; copulatory openings large, sub-rounded, closed to the epigynal posteromargin. Spermatheca globular, each with a width equal to 2/3 of the breadth of booklung cover, separated by a gap measuring around its own diameter; copulatory ducts coiled the base of spermathecae, starting near the rebordered epigynal posteromargin, and ended at the posterolateral margins of spermathecae; fertilization ducts short, and thin, connected with the bases of the spermathecae.

##### Distribution.

Known only from the type locality.

#### Sinanapis wuyi

Taxon classificationAnimaliaAraneaeAnapidae

Jin & Zhang, 2013

Figs 8 , 9

##### Type material.

*Holotype* ♂ (MHBU), CHINA: Fujian Province, Wuyi Mountains, Nankeng, 27°56.000'N, 118°06.000'E, 6.VIII.2010, F. Zhang leg. (examined).

##### Other material examined.

3♂ 3♀ (HNU), CHINA: Hunan Province, Dawei Mountains, 28°14.598'N, 114°03.858'E; 1526 m, 1.V.2012, J.L. Wan leg.

##### Diagnosis.

The male of *S.wuyi* can be distinguished from these of *S.crassitarsa* and *S.medogense* sp. n. by the lack of a rasper-like dentigerous patellar process on the palp, and by having anterior median eyes (Figure 9D; Yuan and Peng 2014: figs 7, 9). In the other two species, the dentigerous patellar process is present, and the anterior median eyes are absent (Figs 1D, 2C, 5G, 6H). It differs from *S.longituba* by having a shorter basal apophysis not exceeding the palpal femoral length, and the shorter copulatory ducts not coiled around the spermathecae (Figure 9A–F). One the other hand, in *S.longituba*, the very long basal apophysis exceed the at least 3 times the length of the palpal femur, and the lengthy copulatory ducts coil around the spermathecae (Figure 4A, B, G, H).

##### Description.

See Figs 8A–H, 9A–F and Jin and Zhang (2013), and Yuan and Peng (2014).

##### Distribution.

China (Hainan, Jiangxi, and Fujian).

**Figure 10.**
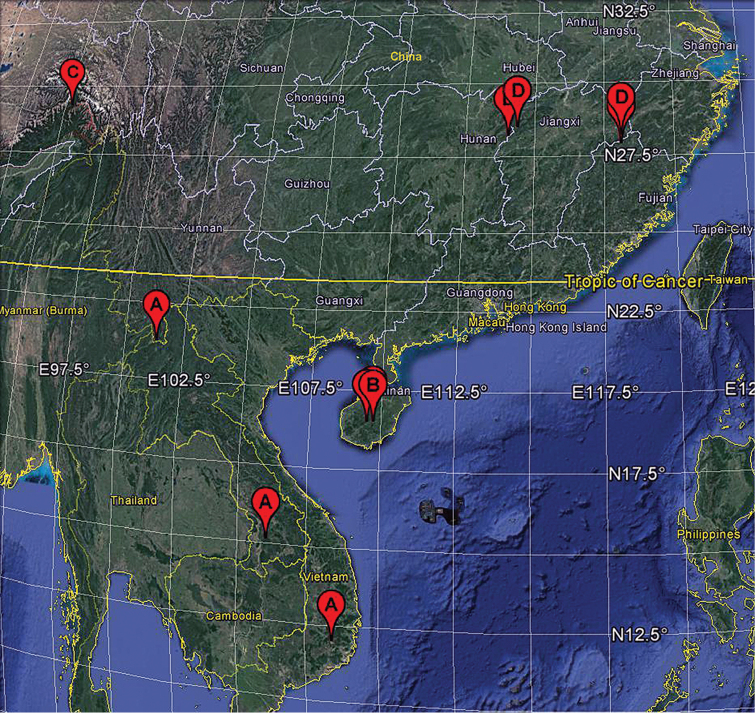
Distribution records of *Sinanapis* spp. in the world. **A***S.crassitarsa* Wunderlich & Song, 1995 **B***S.longituba* Lin & Li, 2012 **C***S.medogense* sp. n. **D***S.wuyi* Jin & Zhang, 2013.

## Supplementary Material

XML Treatment for Sinanapis

XML Treatment for Sinanapis crassitarsa

XML Treatment for Sinanapis longituba

XML Treatment for Sinanapis medogense

XML Treatment for Sinanapis wuyi
